# ‘I can no longer do my work like how I used to’: a mixed methods longitudinal cohort study exploring how informal working mothers balance the requirements of livelihood and safe childcare in South Africa

**DOI:** 10.1186/s12905-021-01425-y

**Published:** 2021-08-06

**Authors:** Christiane Horwood, Rachael Hinton, Lyn Haskins, Silondile Luthuli, Sphindile Mapumulo, Nigel Rollins

**Affiliations:** 1 Centre for Rural Health, Howard College Campus, University of KwaZulu-Natal, Durban, South Africa; 2 RHEdit, Geneva, Switzerland; 3 Department of Maternal, Newborn, Child and Adolescent Health, World Health Organization, Geneva, Switzerland

**Keywords:** Childcare, Workplace, Workplace health, Working women, Informal economy, Maternal health, Child health, South Africa, Africa

## Abstract

**Background:**

Returning to work after childbirth is challenging for working mothers. Childcare quality may have lifelong effects on children’s health, development and cognitive function. Over 60% of working women globally are informal workers without employment or maternity protection, but little is known about how these women care for their children.

**Methods:**

We conducted a mixed-methods longitudinal cohort study among informal women workers in Kwazulu-Natal, South Africa between July 2018 and August 2019. Participants were followed up from late pregnancy until they had returned to work. We conducted structured quantitative interviews and in-depth qualitative interviews at different time points: before and after the baby was born, and after returning to work. Subsequently, a photovoice activity was conducted with groups of participants to explore the childcare environment. We employed narrative thematic analysis for qualitative data and descriptive analysis for quantitative data.

**Results:**

24 women were recruited to participate. Women returned to work soon after the baby was born, often earlier than planned, because of financial responsibilities to provide for the household and new baby. Women had limited childcare choices and most preferred to leave their babies with family members at home, as the most convenient, low cost option. Otherwise, mothers chose paid carers or formal childcare. However, formal childcare was reported to be poor quality, unaffordable and not suited to needs of informal workers. Mothers expressed concern about carers’ reliability and the safety of the childcare environment. Flexibility of informal work allowed some mothers to adapt their work to care for their child themselves, but others were unable to arrange consistent childcare, sometimes leaving the child with unsuitable carers to avoid losing paid work. Mothers were frequently anxious about leaving the child but felt they had no choice as they needed to work.

**Conclusion:**

Mothers in informal work had limited childcare options and children were exposed to unsafe, poor-quality care. Maternity protection for informal workers would support these mothers to stay home longer to care for themselves, their family and their baby. Provision of good quality, affordable childcare would provide stability for mothers and give these vulnerable children the opportunity to thrive.

**Supplementary Information:**

The online version contains supplementary material available at 10.1186/s12905-021-01425-y.

## Background

For working mothers leaving a child in non-parental care is associated with risks, and early return to work may have wide-ranging consequences for both mother and child. Globally, most countries provide formal workers with a period of paid maternity leave. This allows mothers to spend time with their babies which has important benefits for the physical and mental health of the mother, including reducing the risk of post-natal depression [1]. Longer maternity leave is beneficial for the baby’s health, and is associated with reduced infant mortality [1, 2], most likely because of the strong positive association between the duration of maternity leave and breastfeeding [3]. Longer maternity leave is associated with higher rates of breastfeeding initiation, longer periods of exclusive breastfeeding and longer breastfeeding duration [4], all of which are important for child health and development [5], particularly for low-income families who may be unable to afford breastmilk substitutes [6]. Longer duration of maternity leave has been associated with improvements in other determinants of child health, including immunisation coverage and child growth [7, 8].

Childcare arrangements for pre-school children vary widely in terms of the type, quantity and quality of care provided, and have an important influence on child health and development, particularly during the first three years of life, with effects that may last into adulthood [9–11]. High quality childcare is characterised by sensitive interactions between adults and young children, availability of stimulating materials promoting learning, appropriate adult–child ratios, and trained providers. Responsive, developmentally appropriate childcare can enhance child development, leading to children being more confident, with improved social skills and higher learning achievements ahead of school entry, and can contribute to sustained improvement in school performance [12]. Good quality childcare can mitigate against many of the ill effects of poverty and maternal depression [10, 11].

In contrast, studies have shown that poor quality childcare is associated with more behaviour problems, poor language skills and lower educational attainment compared to children receiving high quality childcare [11]. More time spent in care can be associated with less positive engagement between the mother and child, and can lead to insecurity and increased stress among infants and toddlers [13], with negative consequences for young children’s cognitive, behavioural and emotional development and mental health [9, 11, 14]. Interventions to improve childcare have long-term beneficial outcomes including improved competence [15] and wage earning in adulthood [16], providing a strong economic justification for such interventions [14].

Globally about 60% of all working women work in the informal economy, a sector which continues to grow, particularly in low-income settings [17]. Informal workers generally lack the social protection received by formal workers, including access to paid leave, sick leave, maternity leave or unemployment benefits [18]. Informal work comprises diverse work settings and occupations, including informal traders, agricultural workers, and domestic workers, and is associated with poverty and vulnerability. The informal work environment is characterised by poor job security, low earnings and unsafe work conditions, particularly for women [19, 20].

In South Africa (SA) among a total of 6.8 million working women, almost 2 million women work informally [21]. Responsibilities for child care and household work usually fall disproportionally on women, reducing their ability to work [22], and women with children consistently have lower earnings than their male counterparts [23]. Women in the informal economy are vulnerable to losing their jobs if they take leave, so they often return to work soon after childbirth [24]. The need to work has an impact on how mothers in informal work care for their children, particularly affecting where and by whom childcare is provided during working hours [25]. Mothers living in poor areas or with low income or non-standard work schedules are more likely to choose informal care for their children on grounds of cost and convenience. In addition, quality of formal childcare in these areas is often substandard or unsafe so mothers may place more trust in family members [26, 27].However, there are few studies exploring childcare in low income settings [28], and it is important to further understand the choices these mothers make regarding the care for their young children on their return to work.

In this article we present data from a longitudinal cohort study among informal workers in Durban, SA, and describe their experiences of returning to work and leaving their baby in non-parental care or caring for the baby in the work environment. Based on our findings we suggest ways to support these mothers to delay their return to work and improve the quality of childcare in this setting.

## Methods

We undertook a longitudinal cohort mixed-methods study with mothers in informal work between July 2018 and August 2019. In-depth interviews (IDIs) and photovoice activities were used to explore how informal women workers planned, arranged and managed their children’s care on returning to work after childbirth, and how they balanced their responsibilities for childcare and informal work. Employing a longitudinal qualitative design allowed for exploration of participants’ lived experiences of change, capturing the complexities of experience at critical moments. In addition, we collected quantitative data at each time point to describe women’s contexts, plans and practices in order to build a more comprehensive understanding of participants’ situation. Mixed methods designs are particularly suited to exploring complex multi-level processes and systems [29, 30]. The narrative approach was used to guide data collection and analysis. This approach allowed researchers to use the stories told by the women, including through photographs, to explore and understand their lived experiences. Storytelling provided researchers with an in-depth, well-rounded view into people’s lives [31].

### Study site

The study was conducted in two townships areas in Durban, KwaZulu-Natal (KZN). In these areas average earnings are low, with high rates of unemployment and poverty [32]. From 2011 census data, both areas had low levels of education and high unemployment among residents. However, most school age children were in fulltime education and the proportion of homes owned by residents was over 60% in both areas. Although some households had poor service delivery, most received water from a local service provider, over 60% had access to a flush toilet, and most houses were connected to electricity. This represents considerable improvements in service delivery over recent years. Although many houses were formally constructed, informal dwellings or shacks made from plywood, corrugated metal, and sheets of plastic remain fairly common in these areas [32]. In addition, these areas were a designated target for a government urban renewal program, so road and railway infrastructure has been improved, new shopping centers built, and a new hospital is under construction.

Health care is provided free for pregnant women and children in primary health care (PHC) clinics, and antenatal clinic (ANC) attendance is high at 94% [33]. HIV prevalence in KZN is very high: 41.1% of women attending ANC tested HIV positive in 2017 [34]. Mothers or primary caregivers of children with a low-income are entitled to a government Child Support Grant (CSG) of R420 (USD 30) per month for each child [35]. The local language is isiZulu.

### Study participants and recruitment

Based on our experience of undertaking qualitative studies including a previous longitudinal study [36], we estimated that twenty participants would be sufficient to achieve the study aims and reach data saturation. We, therefore, aimed to recruit 24 participants to allow for participant attrition during the study period. For recruitment purposes, an informal worker was defined as someone who worked as an employer or employee in an informal business not registered for value added tax (VAT) or income tax, or who was an own account (self-employed) worker. Informal workers, by definition, did not make contributions, either themselves or via their employer, to SA mandatory unemployment insurance (UIF), and did not have an employment contract. Domestic workers were defined separately as workers who undertook domestic or childcare work in private households and were defined as informal workers regardless of whether they had contracts or made UIF contributions. Women were eligible to participate in the study if they were defined as informal workers, aged 18 years or older and between 32 and 38 weeks pregnant. Women were excluded if they worked less than three days per week, had been in informal work for less than six months, or if they planned to leave the study site before the child was six months old.

Participants were recruited at one ANC clinic in each of the two townships. Women were screened for eligibility by trained field staff and all eligible women were requested to participate. Women interested in participating met with researchers at a convenient location outside of the health facility. Only after a further two field visits, to obtain informed consent and collect baseline data, were participants assigned a study number and formally enrolled in the cohort. The recruitment process aimed to ensure that participants understood the requirements of participating over a long follow-up period, and had adequate opportunity to withdraw from the study.

### Data collection

**Fig. 1:**
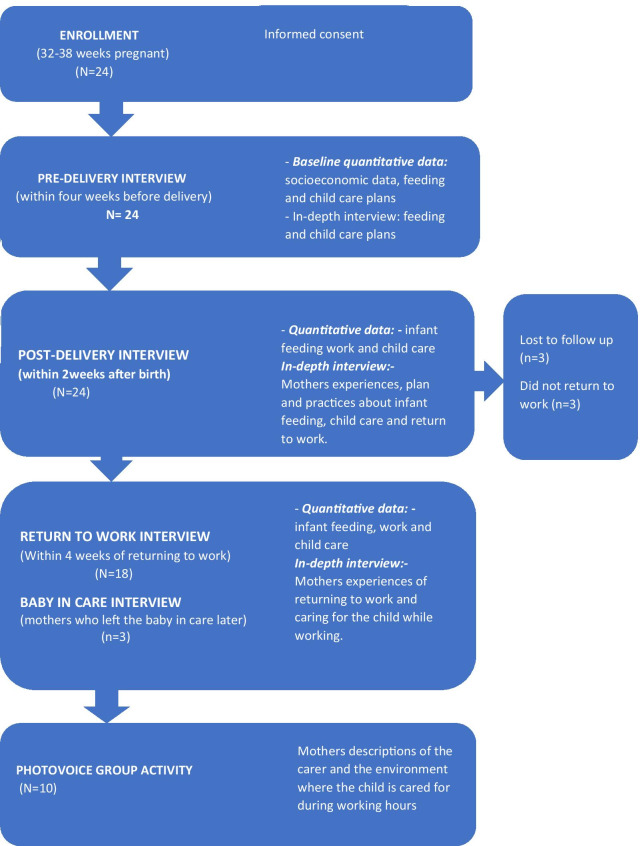
Data collection

Participants were followed up from pregnancy until they had returned to work and left the baby in non-parental care, or for one year, whichever was shorter. A range of data collection methods were used including quantitative questionnaires, IDIs and group photovoice activities, to develop a comprehensive understanding of the research problem. Quantitative and qualitative data were collected pre-delivery, post-delivery, and after returning to work (Fig. 1). Three mothers who took the baby with them on returning to work and left the baby with a carer at a later time had an additional baby-in-care interview. Experienced female researchers (SL, SM) who were trained in qualitative research to masters level, undertook all data collection. Data was collected from each participant by the same researcher throughout, allowing rapport and trust to build up between researchers and participants, enabling participants to voice sensitive concerns and provide detailed and contextualised accounts of their experiences [37].

Interviews were conducted at times and locations convenient for participants in isiZulu or English, depending on the participant’s preference. Researchers contacted participants by telephone every two weeks throughout the study period to maintain contact and monitor participants’ progress towards transition points.

### Quantitative data

Quantitative data collection tools were developed to track the context, plans and practices of mothers at each time point (Supplementary files 1 & 2). Tools were piloted among informal workers not participating in the study, and adapted accordingly. Baseline sociodemographic data, type of informal work, and plans for childcare and return to work, were collected at the pre-delivery interview. During the post-delivery, return to work and baby-in-care interviews, data were collected about the participant’s current work and childcare plans and practices (Fig. 1).

### Qualitative data

*In-depth interviews* IDIs were undertaken to explore participants’ perceptions and experiences of returning to work and providing and arranging childcare at each time point (Fig. 1). Interviews explored how and why mothers’ plans for childcare and return to work changed over time, mothers’ practices caring for the child during work hours, and how they balanced work and childcare responsibilities. Each IDI included a review of previous interviews to maintain a focus on the longitudinal nature of the data.

*Photovoice activity* On completion of all IDIs, all women who had returned to work were invited to participate in a group photovoice activity aimed at further exploring the childcare environment. All participants who were available and willing to participate were included in the photovoice activity. Photovoice uses photographs and storytelling to prompt participants, shifting the focus from the participant to the photograph, making it easier to discuss sensitive subjects. The technique promotes critical dialogue to improve knowledge and understanding of participants’ concerns by empowering them to record and reflect on key parts of their lives [38–40].

Photovoice activities were conducted in local office space and participants were transported to the venue. Participants attended two meetings: a preparatory meeting and a group photovoice discussion. At the preparatory meeting participants were given cameras and instructions to capture pictures that best represented the environment where their child was cared for during working hours. At the second meeting participants selected 4–6 photographs that most represented the childcare environment. These were pinned to the wall and each participant described their photographs to the group, highlighting why the pictures were chosen as well as positive and negative aspects of the childcare environment. On completion of all presentations, participants reflected on and discussed each other’s stories.

### Data analysis

IDIs and photo-voice groups were audio-recorded, transcribed verbatim and translated into English. Quality control was conducted by researchers who listened to a selection of audio-recordings to ensure transcripts were accurate and complete. A sample of transcripts were coded independently by three members of the research team to identify a priori themes based on the research questions, as well as new themes emerging from the data. Consensus on coding was reached after discussion, comparison and refinement of themes. To ensure validity and reliability in the interpretation of the data, the research team met every two weeks to discuss the data analysis. Nvivo 12.3 software was used to support the analysis.

Quantitative data was collected with an electronic tablet using proprietary software and uploaded onto a server in real time. Data was converted into SPSSv26 for analysis. Descriptive data was presented as frequencies only.

## Results

Twenty-four women in informal work were enrolled in the cohort in the last trimester of pregnancy. We present data from 18/24 mothers who returned to work. Six mothers were excluded from this analysis: three mothers lost their jobs and could not find employment so were unable to return to work; and three mothers were lost to follow up before they returned to work (Fig. 1). Two photovoice groups were conducted with five participants each (total 10 participants).

The median age of participants at pre-delivery interview was 28.5 years (SD 4.7; IQR 25.0–30.7) and all women were in a relationship with the baby’s father at baseline. Participating women worked in a variety of informal jobs including as domestic workers (6), informal vendors (1), home-based workers (4), hairdressers (5), fuel attendant (1) and informal tuck shop owner (1). A summary of participants’ sociodemographic characteristics is shown in Table 1.

**Table 1:** Participants’ sociodemographic characteristics (baseline quantitative interview)

Mothers	N = 18
Population group: black	18
South African Zulu	15
South African Xhosa	2
Zimbabwean	1
Relationship status	
Married	1
In a relationship, living with partner	11
In a relationship, not living with partner	6
Mothers household composition	
Partner non-resident, lives with family members ± her children	8
Partner non-resident, lives alone with her children	1
Partner resident, lives alone with partner ± her children	5
Partner resident, lives with other family members ± her children	4
Education	
Secondary schooling: grade 8—11	13
Completed schooling: grade 12	5
Number of children	
None (first pregnancy)	1
1–2	14
3–4	3
Pregnancy was planned	7
Self-reported HIV positive (all on antiretroviral treatment)	7
Receives financial support from father of baby	18
**EMPLOYMENT**	
Type of employment reported by mothers	
Employed	10
Own account (self-employed)	8
Reported monthly income	
< R1000 (< $70)	3
R1000- R3000 ($70- $210)	14
> R3000 (> USD210)	1
**HOUSEHOLD CHARACTERISTICS**	
Type of house	
Formal brick/ cement	15
Informal traditional	1
Informal shack	2
*Main source of drinking water for the household*	
Piped -inside the home	4
Piped – outside the house but on the property	12
Piped – public tap	2
*Type of toilet used by household*	
Flush toilet inside the house	5
Flush toilet outside the house	7
Ventilated pit latrine	6
Toilet is shared with other households	4
Household connected to electricity	18
Household has a working fridge	15

We describe mothers’ plans for child care and return to work, reasons for returning to work early, the reasons for their childcare choices and experiences of different childcare options.

### Plans for returning to work

Before the baby was born, all women planned to take some time off after childbirth: ten mothers planned to return to work before the baby was two months old; six mothers when the baby was three to four months old; and two mothers when the baby was five to six months old. To support themselves during this time mothers planned to use various sources of income including their savings, CSG received for their older children, or support from the child’s father or other family members. A few mothers reported they would continue working from home. Mothers also stated that they would immediately apply for a CSG for the new baby. In practice many mothers returned to work earlier than planned (Table 2). One mother returned to her previous work before the baby was two weeks of age and three mothers were doing casual paid work within two weeks of the baby’s birth.

**Table 2:** Reported return to work and childcare practices (return-to-work quantitative interview)

	N = 18
**Age of child on return to work/months**	
Less than one month	1
1- < 2 months	9
3- < 4 months	3
5- < 6 months	4
Above 6 months	1
**Place where child is cared for during work hours**	
Mother’s own home	5
Carer’s home	3
Creche	3
With mother at workplace	3
With mother working at home	4
**Person who cares for the child during work hours**	
Grandmother	2
Father	1
Other relative	3
Non-relative or creche	5
Mother (at work or at home)	7

Before returning to work, participants carefully considered the benefits and risks of the different childcare options available to them. These included having the baby cared for by a family member or paid carer, leaving the baby in day care (crèche), or caring for the baby themselves, either taking the baby with them to the workplace or working from home. Some mothers also considered sending their baby to their family (parental) home, often in a rural area, to be cared for by grandparents.

I did think about taking her home (mother’s parent's home) but again I thought she was too young and my parents are quite old and there are other children [there] as well. I also considered the crèche but I was uncomfortable with it because you find that there are too many children and this one is still very young…so I ended up taking her to my neighbour’s.’ (SL05, Photovoice group 1)

### Reasons for returning to work

At the time of returning to work, 11 mothers left their baby in non-parental care, three mothers first took their baby to work with them but later left the baby in care, and four mothers worked from home and cared for the baby themselves (Table 2).

The financial pressure of meeting the costs of a new baby and fulfilling financial obligations to the household was the major reason for mothers returning to work. Several women described severe financial pressure, such as being unable to provide necessities, including food, for their older children or pay for household essentials, such as electricity.

It was the financial circumstances [that made me return to work early]. The child needs nappies. We also need money in the house. We were relying on the child support grant money but it is insufficient. There are too many things that are needed. It was the money. (SM12, return-to-work interview)

Some mothers experienced less severe financial constraints, usually because of financial support from family members, but felt a strong responsibility to contribute to the household. These mothers preferred to return to work rather than rely on family members or the child’s father, who also had limited financial resources. In addition, participants reported that earning their own income gave them a strong sense of independence and the freedom to make their own choices.

It is like I have said, that I only earn if I have worked. I work as a dressmaker. If I do not make dresses I do not get any income. The child needs nappies and everything. I cannot be asking for everything from the child’s father. It is better if I can also do what I need to do with the money that I get. (SL05, return-to-work interview)

### Choice of child carer

A common theme expressed by mothers when choosing a carer was that they preferred to leave their baby with an older woman who had childcare experience, and was perceived to be more responsible and knowledgeable about childcare.

It is much better if an elderly person is looking after my baby because she will know what to do if the baby cries or the baby’s temperature rises. When the baby is hungry; she will be able to see that the baby is hungry. So, I decided to ask the granny from next door to look after her, she knows a lot about babies and there is a lot of information that I get from her. (SL01, Photovoice group 1)

The preferred choice for most participants, when available, was a trusted older family member. This was also the most convenient and low-cost option for child care, as in most cases the family member was not paid.

I know that she (child’s grandmother) is someone that I trust. It is not the same as leaving my child with a nanny from outside. I know that she is my parent. So, in everything I am comfortable. There is nothing can hurt my child, or there is nothing bad that she can do to the child. (SL06, return-to-work interview)

**Fig. 2:**
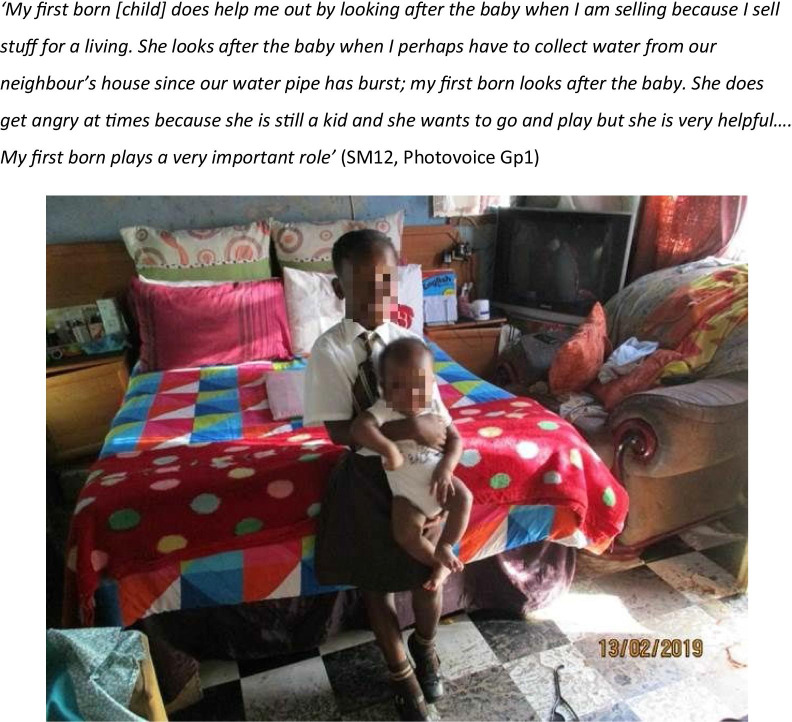
PHOTOVOICE STORY ONE: sibling looks after the baby

Despite the preference for older carers, four mothers reported that their child’s main carer was under 18 years old. In several cases, mothers described how they depended on their older children to care for the baby when they were at work or busy with other household responsibilities (Fig. 2).

**Fig. 3:**
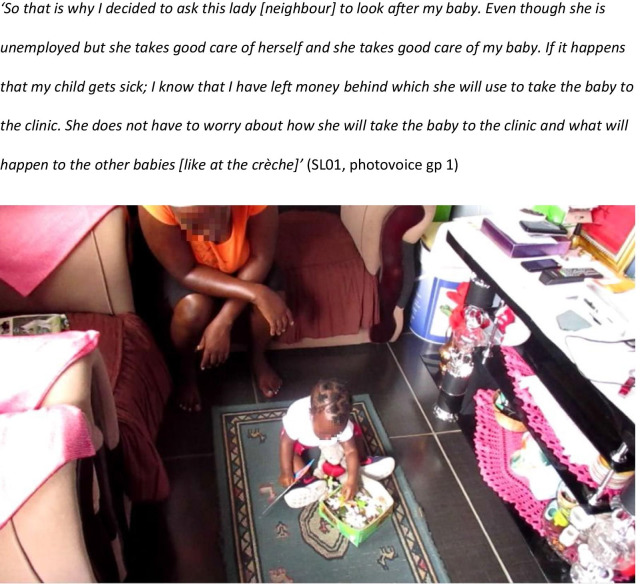
PHOTOVOICE STORY TWO: neighbour cares for the baby

Five mothers chose to leave the child with a paid carer who was not a relative, usually a neighbour. Mothers reported they chose paid carers because they were available, lived nearby and were not working. Childcare experience was an important consideration, and carers who were clean and kept the environment clean were strongly valued by mothers (Fig. 3).

A few mothers expressed concerns that carers sometimes prioritised their own commitments over caregiving responsibilities and could be unreliable. In some instances, mothers were not always certain about who was looking after their child during the day:

The woman that I used to leave my child with earns a living by looking after children. She is not running a crèche but she just looks after children. If she is gone to order stock [for her other business] she leaves the kids with her children. I am not comfortable with my child being looked after by children. (SL01, return-to-work interview)

For a few mothers, the unpredictable nature of informal work made it difficult to establish regular childcare arrangements, so they sometimes had to leave the child with whichever carer was available at the time, even if that person was not a suitable carer.

I had to go and plait this other woman who was sick, so I knew I could not bring my child with me. I asked this other granny to look after my baby. I told her that I will not be able to take my baby to that house…. the granny said I can leave her with her so I left my baby with her. They told me that she was crying so much that day that I had to come back. (SL01, Return-to-work interview)

Three mothers paid for formal childcare in a creche as they felt this was the most reliable option:

Creche is better because that woman (who owns the creche), we sat down and had a discussion, and she said she would look after my child. That woman said that even if my shift started at 13h00, she would look after my child until her father came to fetch her. They treat her like their own child at the crèche. She is the youngest one [laughs]. We drop her off at the crèche at 05h30 and then her father drops me off at work at 06h00 and then he also goes to work. (SL08, Return-to-work interview)

The crèche was usually a higher cost option at approximately R250- 500 per month (US$ 15–30), making it unaffordable for many participants. In addition, crèche hours were often fixed and incompatible with the working hours of informal workers. However, several mothers did suggest that they would consider the crèche when the child was older and needed less care.

### Mother as the carer: working with the baby

Three mothers chose to take the baby to work with them, while four own-account workers were able to adapt their schedule to work from home to care for the child. These four mothers had either been working from home before the baby was born or were able to take on different types of informal work which could be done from home, such as laundry and hairdressing.

Everything is returning to normal. The only difference is that I no longer go out to people. They come here. I used to deliver for them. I no longer deliver. The people come here to fetch their stuff. (SL11, Return-to-work interview)

**Fig. 4:**
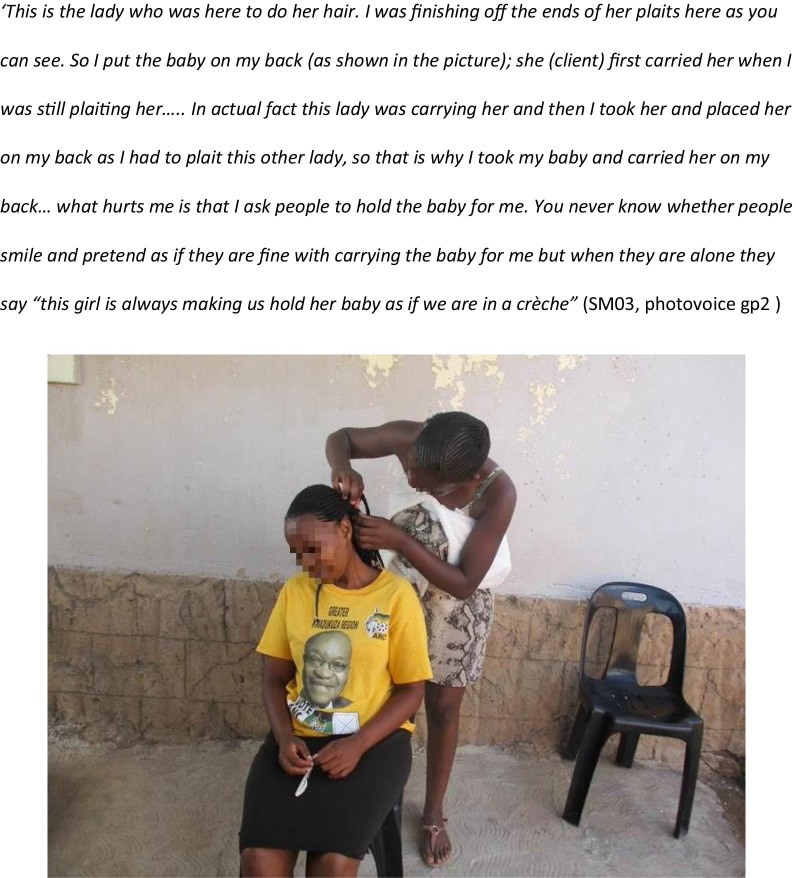
PHOTOVOICE STORY THREE: taking the baby to work

The main concern expressed by mothers taking the child to work was balancing childcare needs with work commitments. Although these mothers had flexibility in caring for their child, it was a challenge to manage the dual commitments (Fig. 4). Many participants mentioned that they were unable to maintain the same amount of work as they did before having their baby, which resulted in a loss of income.

There is change because I can no longer work for the whole day or full time. I only do my work when I have time maybe when the child is sleeping. I try to work quickly. But when the child wakes up I cannot continue working. So, I stop and the work piles up. (SL05, Return-to-work interview)

#### Childcare environment

Most mothers preferred that their child be cared for in their own home. It was a familiar environment, and mothers could avoid the stress of dropping their child at another location before starting work. Mothers who left the child with a carer expressed that having to prepare the child added more time pressure to their day and at times made them late for work.

It is also time consuming to pack his bag and take him there (to the carer), especially if he is agitated and crying. I have to wait for him to calm down and spend time waiting there instead of selling. I lose some money in the process. (SL12, Baby-in-care interview)

Caring for the child in the family home meant that family members were often available in the house to oversee the activities of the carer.

I prefer that [the] carer look after her at home. My sister is also there anyway. She is unemployed. She is always there. She is older and she can tell if the child is hungry and she tells the carer what to do. The carer does as she is told. If she cannot find certain things, she [sister] shows her [carer] because she is always around’. (Sl12, photovoice group2)

**Fig. 5:**
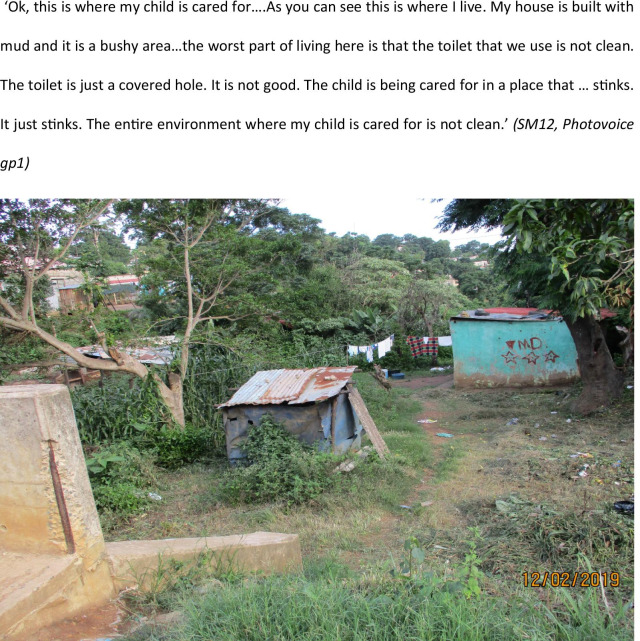
PHOTOVOICE STORY FOUR: where my child is cared for

However, mothers expressed many concerns about the childcare environment, regardless of where the child was cared for, even when it was their own home. Mothers frequently described the childcare environment as hazardous because of poor hygiene facilities, as well as proximity to roads or other environmental hazards (Fig. 5). Several children were cared for in environments with inadequate access to clean water and safe toilet facilities (Photovoice story 4).

The crèche environment was also reported by many mothers as being unsafe with poor quality care, particularly for a young baby. Many mothers considered taking the baby to a creche but rejected this because of concerns about the environment.

The first crèche I went to is on the side of the road and the person who looks after the children looks; how can I say this? She looks like she does not take care of herself. The day I went there to inspect what the place is like I saw the other children who were left by their mothers. One child was eating and the cat there took the meat from the baby. (SL05, Photovoice group1)

Many of the mothers worried that if their child was not watched at all times they could be injured, and described the anxiety they experienced at leaving their child in hazardous environment, particularly when they first returned to work.

I am thinking about the child most of the time. My mind is not where I am. My mind is preoccupied with the child. So, it is not alright. However, there is nothing that we can do. One has to work. (SM12, Return-to-work interview)

The three mothers who took their child to work were also very concerned about exposing their young child to poor safety and hygiene conditions within the work environment:

If you are going to be caring for a small child and also be busy with other things on the side you are prone to making a lot of mistakes. A lot can happen, especially since I put her to sleep inside the shop, anything could fall from the shelves and fall on her and hurt her. Those are the things that I have thought about, and the [poor] air quality in the shop as well. (SL11, Return-to-work interview)

## Discussion

Our findings describe how the vulnerability of informal women workers affected their childcare practices. Participants were low paid and without maternity protection, relying on savings, government grants, and family support to provide for themselves, their family and their newborn baby. As a result of financial pressure, many women returned to work when the baby was very young, and had to choose between various unsatisfactory and unsafe childcare arrangements, contributing to mothers’ anxiety. Women usually relied on informal childcare that was easily available at low-cost, but where the environment was often unhygienic and hazardous, putting the baby’s health at risk. Formal childcare was reported to be of poor quality, unaffordable and not suited to needs of informal workers. Although the flexibility of informal work allowed some mothers to adapt their work conditions to continue working while caring for and breastfeeding the child, this often resulted in reduced earnings. Thus, mothers were constantly juggling the conflicting needs to provide for their household and family, and care for their child.

Childbirth was a vulnerable time for participants as their work was often unstable and insecure. Three mothers lost their jobs as a result and were unable to find a new job leading to substantial hardship. Literature suggests that the disproportionate burden of child and household care shouldered by women not only prevents mothers from working but also is a barrier to seeking work [23]. In most settings it is women who take time off work to provide childcare, or girls who sacrifice their education to help out at home [41]. In our study, several mothers mentioned that their older children played an important role in childcare, and four mothers reported that the child’s primary carer was under 18 years old. The availability of female relatives to provide care, has an impact on the access to child care [23]. Studies in Southern Africa highlight the critical role played by the extended family and wider community in childcare, where it is common for older children, particularly girls, to look after younger children taking them away from their own educational and leisure activities [42].

Quality of childcare is a crucial determinant of child health outcomes, contributing to children’s social, language and cognitive development during early childhood. Interactions with adults are very important for child development, so stability of the carer as well as quality of childcare is critical [11]. It has been suggested that women with young children may choose informal work because it provides flexibility allowing them to work and care for their children, and may provide access to informal support systems unavailable to formal workers [43, 44]. However, flexible work may also be unpredictable, making it difficult for mothers to make fixed childcare arrangements. A few mothers described having to make difficult childcare choices to avoid losing opportunities for paid work, sometimes using inappropriate carers who lacked basic childcare skills. Further, it is likely that using ad hoc carers led to poor interactions and bonding with carers. For children of informal workers to have a good start in life, it is important that affordable, good quality childcare be available. Children in higher quality childcare have better cognitive, language and social developmental outcomes [11, 12]. Poor access to quality early childcare only serves to increase inequalities, and is particularly important for children in low resource settings as they typically have fewer resources for learning in the home and in the community.

Poor quality childcare is a concern in many low-income countries globally; a survey from 31 developing countries revealed the shortage of available child care alternatives for working women [45]. The provision of good quality, accessible, public childcare services is a key policy intervention which has the potential to improve the productivity and incomes of informally working women [22]. In addition, a flexible system is required that enables parents to choose a mix between formal and informal care should they prefer [46]. However, the provision of state-based childcare for working women with children has generally not been considered a priority by governments who have focussed on other development goals [22]. A review of childcare in the global south shows that policies often fail to take account of working women’s need for childcare and this has had a negative effect on women’s employment [22]. Universal quality childcare services are among the most effective tools for supporting the labour force participation of women with young children, including in low- and middle-income countries [47].

Study participants demonstrated some understanding of factors that contribute to high quality childcare, mentioning practical, nutritional, hygiene and safety considerations. However, they did not express concern about activities to facilitate children’s cognitive and socioemotional development, access to developmentally appropriate materials, or the level of stimulation and interaction between carers and children. Although we acknowledge that it is appropriate that nutrition and safety are mother’s primary concerns, we suggest that there could be a role for improving awareness about the importance of early child development both at home and in the childcare setting. Children’s development requires nurturing care, characterised by an environment that is sensitive to health and nutrition, as well as being responsive, emotionally supportive and developmentally stimulating [14], and studies suggest that nurturing care can mitigate against the negative impact of adversities experienced in early life [48]. Interventions to improve mother’s knowledge and the knowledge of the broader community about nurturing childcare could increase awareness of the importance of childcare quality for ensuring that children reach their potential. Nurturing care can be provided without additional resources or with simple low-cost items found at home [49]. Community health workers deployed in communities in SA have the responsibility to visit homes with young children, and could take a strong role in promoting nurturing care [50].

Formal maternity protections safeguard mothers’ time away from work, allowing them to recover from childbirth, breastfeed, be close to their infants and follow their childcare plans. Maternity protection improves infant feeding practices and outcomes for mothers and babies [2, 3]. Women workers in this study did not benefit from formal employment-related protections, and as a result, worked far into their pregnancy or started working soon after childbirth, often earlier than planned, to fulfil their financial obligations. Many participants highlighted the importance of the CSG in supporting the family, supporting findings of a recent SA study among informal workers that the CSG plays an important role in supporting time away from work after childbirth [51]. The CSG is the largest unconditional cash transfer programme in Africa, reaching over 11 million children [52], and has been shown to give important financial support assisting families to provide for children’s basic needs [53]. However, currently CSG application only starts after the baby is born, and the CSG therefore fails to provide financial support during the crucial weeks after childbirth when the mother needs to stay at home with her baby. A maternity grant for informal workers would allow mothers to stay at home longer to care for and breastfeed their infants, and would be an investment in providing these vulnerable children with a better start in life. In SA, the existing CSG could be extended to include the last trimester of pregnancy.

### Study strengths and limitations

The robust qualitative methodology employed in this study enabled in-depth insights into womens’ experiences over time. However, the qualitative methodology aimed to develop an in-depth understanding of the topic, and is not suitable for generalisation to the wider population of informal women workers.

## Conclusion

Juggling the demands of informal work with many other unpaid demands on women’s time was a significant challenge for informal working women in this study. The continuing gender segregation in unpaid care and domestic work points to the need to address the underlying social norms related to gender roles in SA and elsewhere [47]. Women’s access to income-earning activities, and therefore their economic rights, were affected by their role as carers and their household responsibilities. Provision of child care and social protection would allow women to return to work safely, reduce maternal stress, with multiple benefits for care and development for children. A transformative social protection system is needed to take account of the needs of children and their caregivers, and provide a policy framework which looks to address the vulnerabilities experienced by women and their children.

## Supplementary Information

**Additional file 1.** Baseline quantitative questionnaire.

**Additional file 2.** Follow up quantitative questionnaire.

## Data Availability

All data, transcripts and study tools to support the findings of this study are available from the Centre for Rural Health and will be made available upon reasonable request from the principal investigator or corresponding author. The data is not available publicly because analysis is ongoing and further manuscripts are being written.

## Abbreviations

ANC: Antenatal clinic
CSG: Child support grant
IDI: In-depth interview
IQR: Inter-quartile range
KZN: KwaZulu-Natal
SA: South Africa
SD: Standard deviation
UIF: Unemployment insurance fund
USD: United States dollar
