# Supplementary material for: ‘I can no longer do my work like how I used to’: a mixed methods longitudinal cohort study exploring how informal working mothers balance the requirements of livelihood and safe childcare in South Africa
Source: BMC Womens Health. 2021 Aug 6;21:288. doi: 10.1186/s12905-021-01425-y (PMC8349013; doi:10.1186/s12905-021-01425-y)
Supplement: Supplementary file 1 — Additional file 1. Baseline quantitative questionnaire. [file 12905_2021_1425_MOESM1_ESM.pdf]

# LINCs Baseline questionnaire

**The baseline questionnaire consists of the following sections**

1. **Administration and informed consent**
2. **Mothers information**
3. **Fathers information**
4. **Household information**
5. **Health services and plans for work and the baby**
6. **Postnatal depression scale (separate sheet)**
7. **Food security (separate sheet)**

| **Section 1: Administration and Informed consent** | | | | | | | | | |
|---|---|---|---|---|---|---|---|---|---|
| 1.1 | Was informed consent obtained?<br>**Uchazeliwe, imvume itholakele?** | 1 = Yes | | | | 2 = No, participant refused to participate<br>➔ ***End interview*** | | | |
| 1.2 | Site of recruitment<br>**Indawo la kuxoxisanwa khona** | | | | | | | | |
| 1.3 | Tracking number<br>**Inombolo yokulandelela** | | | | | | | | |
| 1.4 | Interviewers name / initials<br>**Igama lomphenyi mibuzo/ Inishiyali** | | | | | | | | |
| 1.5 | Date of interview<br>**Usuku lwenxoxo mibuzo** | D | D | M | M | Y | Y | Y | Y |

## Section 2: Mothers information

| I would like to start by asking you some questions about yourself.<br>**Ngizoqala ngokukubuza imibuzo emayelana nawe** | | | | | | | | | |
|---|---|---|---|---|---|---|---|---|---|
| M2.1 | What is your date of birth?<br>**Ingabe wazalwa nini?** | D | D | M | M | Y | Y | Y | Y |
| M2.2 | Mothers population group (observe)<br>**Ubuhlanga bukamama (bheka)** | 1 = African | | | | | | | |
| | | 2 = Indian | | | | | | | |
| | | 3 = Colored | | | | | | | |
| | | 4 = White | | | | | | | |
| | | 5 = Other | | | | | | | |
| M2.3 | Mothers most recent relationship status?<br>**Ingabe ukhona umuntu ozwana naye?** | 1 = Single | | | | | | | |
| | | 2 = Married | | | | | | | |
| | | 3 = Separated / divorced / widowed | | | | | | | |
| | | 4 = In a relationship and living with partner | | | | | | | |
| | | 5 = In a relationship and not living with partner | | | | | | | |
| M2.4 | What is the highest grade you passed at school?<br>**Ingabe iliphi ibanga oliphumelele esikholeni?** | 1 = Never attended school | | | | | | | |
| | | 2 = Primary school: grade 1 to grade 7 | | | | | | | |
| | | 3 = Secondary school: grade 8 to grade 11 | | | | | | | |
| | | 4 = Completed schooling: grade 12 | | | | | | | |

| | | | |
|---|---|---|---|
| M2.5 | Is this your first pregnancy?<br>**Ingabe uyaqala ukukhulelwa?** | 1 = Yes ➔ ***Skip to 2.8*** | 0 = No |
| M2.6 | How many other biological children do you have? (not counting this pregnancy)<br>**Bangaki abanye abantwana onabo abazalwa nguwe (uma singamubali lo omthwele?** | ………………………. Other children | |
| M2.7 | How many of your biological children are staying in the same household as you (at least four nights per week)<br>**Bangaki abantwana abazalwa nguwe ohlala nabo endlini (okungenani ubusuku obune esontweni)?** | …………………………. Children | |
| M2.8 | Did you intend/plan/try to become pregnant with this baby?<br>**Ingabe wawukuhlosile / ukuhlelile/ ubuzama ukukhulelwa lomntwana?** | 1 = Yes | 0 = No |

## Section 3: father's information

| | | | |
|---|---|---|---|
| Now I would like to ask you for information about the father of your baby<br>**Ngicela ukubuza imibuzo mayelana nobaba womntwana.** | | | |
| F3.1 | How old is the baby's father?<br>**Uneminyaka emingaki ubaba womntwana?**<br>***Record 99 if mother does not know*** | ………………………………………….Years | |
| F3.2 | Are you still in contact with the father of your baby?<br>**Ingabe nisaxhumana nobaba wontwana?** | 1 = Yes | 0=No ➔***Skip to 3.5*** |
| F3.3 | Are you still in a relationship with the father of your baby?<br>**Ngabe usenabo ubudlelwano nobaba womntwana?** | 1 = Yes | 0=No ➔ ***Skip to 3.5*** |
| F3.4 | Are you staying in the same house as the father of your baby most nights? ***4 nights or more a week***<br>**Ngabe uhlala naye ubaba womntwana? Ngesonto ubusuku obune noma ngaphezulu** | 1 = Yes | 0=No |
| F3.5 | What is the highest grade the father passed in school?<br>**Ingabe eliphi ibanga eliphezulu elaphaswa ubaba womntawana eskoleni?** | 1 = Never attended school<br>2 = Primary school: grade 1 to grade 7<br>3 = Secondary school: grade 8 to grade 11<br>4 = Completed schooling: grade 12<br>5 = Do not know | |
| | | | |

| | | | |
|---|---|---|---|
| F3.6 | Does the father of your baby work?<br>**Uyasebenza ubaba womntwana?** | 1 = Yes | |
| | | 0 = No ➔ ***Skip to 3.9*** | |
| | | -1 = Do not know<br>➔ ***Skip to 3.9*** | |
| F3.7 | What work does the baby's father do?<br>**Ingabe ubaba wontwana wenza msebenzi muni?** | | |
| F3.8 | Is the father of your baby in formal employment or informal employment?<br>**Ingabe usebenza kuphi ubaba ka(gama)?**<br>*Formal employment means receives payslips, pays UIF, paid holiday, has a contract etc* | 1= Formal employment | |
| | | 2 = Informal employment | |
| | | -1 = Do not know/ unsure | |
| F3.9 | Has the baby's father given you any money to take care of you while you have been pregnant?<br>**Ngabe ubaba wontwana useke wakunikeza imali ukuze uzinakekele njengoba usukhulelwe?** | 1 = Yes | 0 = No |

## Section 4: Household information

| | | |
|---|---|---|
| Now I would like to ask for information about the household you are living in **(at least 4 nights per week).** (household = social unit composed of those living together in the same dwelling who usually cook together). This may comprise a whole building/part of building/several buildings<br>**Need to be sleeping there at least 4nights a week**<br><br>**Manje ngicela ukukubuza imibuzo mayelana nendlu ohlala kuyona.**<br><br>**(Indlu ohlala kuyona – ilapha kunabantu abantu abahlala ndawonye esakhiweni futhi nipheka ndawonye kuleso sakhiwo). Lokhu kungaba indlu ephele / eyinxenye yesakhiwo/ izakhiwo eziningi. Kundingeka kube ukuthi ulala khona okungenani ubusuku obune ngesonto** | | |
| H4.1 | How long have you been living in the household where you are currently staying?<br>**Usuhlale kulomuzi ohlala kuwona isikhathi esingakanani?** | 1= Less than 1 year<br>2= 1-2 years<br>3 = 3 years or more |
| H4.2 | How many adult (12 years or over) people are living in the household with you?<br>**Bangaki abantu abadala (abangaphezulu kweminyaka ewu-12) ohlala nabo kulomuzi ohlala kuwona?** | …………………………………(Adults) |
| H4.3 | How many children (under 12 years) are living in the household with you?<br>**Zingaki izingane (ezingaphansi kweminyaka ewu-12) ezihlala nawe kulomuzi okuwona?** | …………………………………(Children) |
| H4.4 | How many rooms are there in the house where you live?<br>**Unamagumbi amangaki umuzi ohlala kuwo?** | ……………………………… rooms |

| | | | |
|---|---|---|---|
| H4.5 | Who is living in the household with you?<br>**Tick all that apply**<br><br>**Ubani omunye ohlala naye endlini?**<br><br>**Khetha lokho okuhambiselana naye** | 1 = Your mother (child's grandmother) | |
| | | 2 = Your siblings | |
| | | 3 = Other family members | |
| | | 4 = Your other biological children | |
| | | 5 = Non family members | |
| | | 6= Father of the baby | |
| H4.6 | What type of house do you live in?<br><br>**Ingabe uhlala endlini enjani ngesakhiwo?** | 1= Formal brick/cement | |
| | | 2= Informal traditional | |
| | | 3= Informal shack | |
| H4.7 | What is the MAIN source of drinking water for this household?<br>***ONLY ONE RESPONSE ALLOWED***<br><br>**Niwakhaphi amanzi okuphuza?**<br>***IMPENDULO EYODWA EVUMELEKILE*** | 1 = Piped – Inside the house | |
| | | 2 = Piped – Outside but in own yard | |
| | | 3 = Piped – public tap | |
| | | 4 = River water / dam / lake / pond | |
| | | 5 – Tank water (Jojo) / rain water | |
| | | 6 = Borehole | |
| | | 7 = Spring surface water | |
| | | 8 = Tanker truck | |
| H4.8 | What type of toilet is used by the household?<br><br>**Nisebenzisa hlobo luni lwendlu yangasese?**<br><br>***ONLY ONE RESPONSE ALLOWED*** | 1 = Flush toilet inside ➔**SKIP to 4.11** | |
| | | 2 = Flush toilet outside | |
| | | 3 = Ventilated pit latrine (VIP) | |
| | | 4 = Pit latrine | |
| | | 5 = Bucket toilet | |
| | | 6 = Bush / veld / no toilet | |
| H4.9 | Do you share the toilet with other households?<br>**Kukhona nabanye abasebenzisa leyondlu yangasese** | 1 = Yes | 0 = No<br>➔**SKIP to 4.11** |
| H4.10 | How many other households share the toilet?<br>**Mingaki imizi enisebenzisa nayo indlu yangasese?** | ....................................... households | |
| H4.11 | Is the household connected to electricity?<br>**Umuzi unawo ugesi?** | 1 = Yes | 0 = No |
| H4.12 | Is there a working fridge at home?<br>**Ingabe likhona ifriji elisebenzayo ekhaya?** | 1= Yes | 0= No |
| H4.13 | What is the MAIN source of fuel used for cooking food?<br>**Nibasa ngani uma nipheka?**<br><br>***ONLY ONE RESPONSE ALLOWED*** | 1 = Electricity | |
| | | 2 = Gas | |
| | | 3 = Coal | |
| | | 4 = Wood | |
| | | 5 = Cow dung | |
| | | 6 = Paraffin | |

## Section 5: Health service and plans for work and the baby

| | | | | |
|---|---|---|---|---|
| Now I would like to ask you questions about your own health, the health services you use and planning for this baby<br>**Manje ngizokubuza imibuzo mayelana nempilo yakho kanye ne** | | | | |
| WPB5.1 | When is your baby due?<br>**Ingabe ingane yakho izozalwa nini?** | ………………………………Date | | |
| WPB5.2 | How many times have you attended the antenatal clinic for this pregnancy<br>**Usuye kangaki emtholampilo wabakhulelwe?** | ………………………….. times | | |
| WPB5.3 | When you are attending ANC what arrangements do you make with your employer / work?<br>**Uma ugula noma ungakwazi ukusebenza (e.g. uyoxukuza) imaphi amalungiselelo owenzayo?** | 1= Do not go to work<br>2= Arrange someone else to do the work<br>3= Go on a day when I am not working<br>4= On maternity leave<br>5= Other, specify | | |
| WPB5.4 | Specify if other selected or make notes:<br>**Cacisa noma ubhale kabanzi uma kukhethwe okunye** | | | |
| WPB5.5 | Do you get paid if you are unable to work because you are attending ANC?<br>**Ingabe uyaholelwa uma ungakwazi ukusebenza ngoba ugula?** | 1= Yes | 0= no | -1= N/A |
| WPB5.6 | Are you planning to take time off / stop working before you have your baby?<br>**Uhlele ukuthi uzohlala ekhaya noma uzoyeka ukusebenza ngaphambi kokubeletha umntwana?** | 1 = Yes | 0 = No | 2= Currently on maternity leave<br>➔***Skip 5.8*** |
| WPB5.7 | When do you plan to stop work before you have your baby?<br>**Uhlele ukuthi uzoyeka ukusebenza ngaphambi kokuthi ube nengane?** | …………………………months of pregnancy | | |
| WPB5.8 | How are you planning to feed your baby after the baby is born?<br>**Uhlele ukuthi uzomupha ini umntwana uma usutetile?** | 1 = Breastfeed<br>2 = Formula feed ➔***Skip 5.10***<br>3 = Mixed feed (Breast milk and other foods) | | |
| WPB5.9 | How long are you planning to breastfeed?<br>**Uhlele ukuthi uzoncelisa isikhathi esingakanani?** | …………………………… months | | |
| WPB5.10 | Do you plan to go back to work after you have had your baby?<br>**Uhlele ukuthi uzobuyela emsebenzini uma usutetile umntwana?** | 1 = Yes | 0 = No<br>➔ ***End interview*** | |
| WPB5.11 | When do you plan to go back to work after you have had your baby? *(Age of the baby)*<br>**Uhlele ukubuyela nini emsebenzini uma usutetile? *(iminyaka yengane)*** | ………………………………(months) | | |

| | | | | |
|---|---|---|---|---|
| WPB5.12 | Who will look after your baby when you go back to work?<br>**Ubani ozokubhekela umntwana wakho uma usubuyele emsebenzini?** | 1= Child's grandmother | | |
| | | 2= Child's father | | |
| | | 3= Child's sibling | | |
| | | 4= Other relative | | |
| | | 5= Non relative | | |
| | | 6= Yourself (take child to work/work at home | | |
| WPB5.13 | How do you plan to support yourself when you stop working?<br>**Uzozondla ngani uma usuyekile ukusebenza?**<br><br>***(More than one option is allowed)*** | 1 = Savings | | |
| | | 2 = Child support grant from other children | | |
| | | 3 = Disability grant | | |
| | | 4 = Money/in kind support from family members | | |
| | | 5 = Money/in-kind support from the baby's father | | |
| | | 6 = Other (specify) | | |
| WPB5.14 | Specify if other selected or make notes:<br>**Cacisa noma ubhale kabanzi uma kukhethwe okunye** | | | |
| Now I would like to ask you questions about HIV. Please remember that everything you tell me is confidential and that you do not have to answer any question that you do not choose to answer. Please just tell me that you prefer not to answer and I will move onto the next question<br><br>**Manje ngizokubuza imibuzo ephathelene negciwne lesandulela ngculazi. Khumbula ukuthi konke ongitshela kona kuyimfihlo futh akuphoqile ukuthi uphendule noma iyiphi imibuzo ongafuni ukuyiphendula. Ngicela nje ungitshele ukuthi awufuni ukuwuphendula ngobe sengidlulela kumbuzo olandelaya.** | | | | |
| HIV5.15 | Have you been tested for HIV<br><br>**Usuke wahlolela igciwane lesandulela ngculazi?** | 1 = Yes | 0 = No<br>➔***Skip to 6.1*** | -3 = Chooses not to answer<br>➔***Skip to 6.1*** |
| HIV5.16 | What was the result?<br>**Yayithini imiphumela?** | 1 = Positive | 0 = Negative<br>➔***Skip to 6.1*** | -3 = Chooses not to answer<br>➔***Skip to 6.1*** |
| HIV5.17 | Are you currently taking ante-retroviral treatment (ART)?<br>**Njengamanje uyayidla imishanguzo?** | 1 = Yes | 0 = No | -3 = Chooses not to answer |

## Section 6: Work information

| | | | |
|---|---|---|---|
| The following questions are about the work that you are currently doing and the work environment. (includes work environment where usually works if she is on maternity leave)<br>**Imibuzo elandelayo imayelana nomsebenzi owenzayo kanye nendawo osebenzela kuyona ( kubala nendawo osebenza kuyona uma ukwi – maternity leave)** | | | |
| W6.1 | What type of work do you do currently?<br>**Iluphi uhlobo lomsebenzi owenzayo njengamanje?**<br>***(If two jobs select MAIN job)***<br>*Market trader is someone who* ***has a structure*** *provided for them to work at e.g. shelter or table, & trader pays rent. Street vendor is someone who works on the side of the road or in a train station/taxi rank etc. but sets up the stall themselves with no structure available*<br>*Any person making goods for sale and then selling them is a street/market trader* | 1= waste picker<br>2= street or informal vendor (cooking)<br>3= street or informal vendor (non-cooking)<br>4= domestic worker<br>5= market traders<br>6= carry goods for other people<br>7= home based worker (includes making goods for sale)<br>8= other (specify) | |
| W6.2 | Specify if other selected or make notes:<br>**Cacisa noma ubhale kabanzi uma kukhethwe - okunye** | Write answer | |
| W6.3 | How long have you been doing this work? (main occupation)<br>**Sekuyisikhathi esingakanani wenza lomsebenzi (lo owenza kakhulu)?** | 1= less than one year<br>2= One to three years<br>3= Four years or more | |
| W6.4 | How many days do you usually work each week?<br>**Zingaki izinsuku ozisebenzayo evikini?**<br>*Prompt if uncertain*<br>*Mukhuthaze ngemibuzo uma engenaso isiqiniseko* | 1= 3-4 days<br>2= 5-6 days<br>3= 7 days | |
| W6.5 | How would best describe your working condition? | 1= Employee (receives money from employer)<br>2= Own account worker<br>3= Not paid/paid inkind | |
| W6.6 | How much do you earn every month?<br>**Ingabe uhola malini ngenyanga?** | 1= Less than R1000<br>2= 1000-3000<br>3= More than 3000 | |
| Now I would like to ask you questions about your expenses that you have to pay daily, weekly or monthly with the money you earn. All expenses not just work expenses. Please reply by saying Yes or No | | | |
| W6.7 | Do you have to pay for electricity?<br>**Ingabe uyawukhokhela ugesi?** | 1= Yes | 0= No |

| | | | |
|---|---|---|---|
| W6.8 | Do you have to pay for water?<br><br>**Ingabe uyawakhokhela amanzi?** | 1= Yes | 0= No |
| W6.9 | Do you have to pay rent?<br><br>**Ingabe uyayikhokhe irenti?** | 1= Yes | 0= No |
| W6.10 | Do you have to pay for childcare/school fees?<br><br>**Ingabe uyamukhekhela umntwana imali yasenkulisa noma yesikole?** | 1= Yes | 0= No |
| W6.11 | Do you have to pay for food?<br><br>**Ingabe uyakukhokhela ukudla?** | 1= Yes | 0= No |
| W6.12 | Do you have to pay for transport?<br><br>**Ingabe uyayikhokhela i-transport?** | 1= Yes | 0= No |
| W6.13 | Is there anything else you have to pay for?<br><br>**Ingabe kukhona okunye okukhokhelayo?** | 1= Yes | 0= No |
| W6.14 | If Yes, specify<br><br>**Cacisa uma kukhona okunye okukhokhelayo** | | |
