# Supplementary material for: ‘I can no longer do my work like how I used to’: a mixed methods longitudinal cohort study exploring how informal working mothers balance the requirements of livelihood and safe childcare in South Africa
Source: BMC Womens Health. 2021 Aug 6;21:288. doi: 10.1186/s12905-021-01425-y (PMC8349013; doi:10.1186/s12905-021-01425-y)
Supplement: Supplementary file 2 — Additional file 2. Follow up quantitative questionnaire. [file 12905_2021_1425_MOESM2_ESM.pdf]

Section 15: Childcare

To be completed when a mother tells you she is leaving the baby in the care of another person while she is at work			
15.1	Who takes cares of your baby while you are at work?<br>**Ubani onakekela umntwana wakho ngesikhathi usemsebenzini?**	1 = Childs grandmother	
		2 = Childs father	
		3 =Childs sibling	
		4 = Other relative	
		5 = Non relative or crèche	
15.2	Does the baby live with the carer? (baby sleeps there at least four nights / week)<br>**Ingabe umntwana uhlala nomuntu omnakekelayo? (umntwana ulala khona okungenani ubusuku obune ngesonto)**	1 = Yes	0 = No
15.3	Is the carer under the age of 18 years?<br>**Ingabe umuntu onakekela umntwana uneminyak engaphansi kuka – 18?**	1 = Yes	0 = No
15.4	Where is your baby cared for while you are at work?<br><br>**Ingabe unakekelwa kuphi umntwana wakho ngenkathi usemsebenzini?**	1= At your current residence (where you sleep at least four nights/week)	
		2 = At the carers home	
		3 = At a crèche	
		4= Other (specify)	
15.5	What is the **drinking water source at the place where the child is cared for** while you are working (this could be your own home, the carers home or the crèche)?<br><br>**Ingabe lapho anakekelwa khona umntwana wakho ngenkathi usasemsebenzini atholakala kuphi amanzi (lokhu kungaba isemsebenzini wakho, ekhaya lakho, ekhaya la lo omgadayo noma enkulisa)?**	1 = Piped – inside or in the yard	
		2 = Piped – outside the yard	
		3 = Other (specify)	
		4 = Do not know ➔ ***Skip to***	
15.6	Is anything done to the water to make it safe?<br>**Ingabe ikhona into eyenziwayo emanzini ukuthi aphephe?**	1 = Yes	0 = No ➔ ***Skip to***
15.7	What is done to the water to make it safe?<br>**Yini eyenziwayo ukuwenza lawomanzi ukuthi aphephe?**	1 = Boil	
		2 = Add bleach / chlorine	
		3 = Strain through a cloth	
		4 = Use water filter / ceramic / sand / composite	
		5 = Solar disinfectant / leave in the sun	
		6 = Let it sand and settle	
15.8	What type of **toilet is used where the child is cared** for while you are working (this could be your own home, the carer's home or the crèche)?<br>**Ingabe luhlobo luni lwendlu yangasese olusetshenziswayo la ingane inakekelwa khona ngenkathi usasemsebenzini (lokhu kungaba indawo la wena osebenzela khona, ikhaya lakho, ikhaya lombheki wakhe noma inkulisa)?**	1 = Flush toilet inside	
		2 = Flush toilet outside	
		3 = Ventilated pit latrine	
		4 = Pit latrine	
		5 = Bucket toilet	
		6 = Bush / veld / no toilet	

15.9	How are **nappies disposed of where the child is cared** for while you are working (this could be your own home, the carer's home or the crèche)? **Ingabe alahlwa kanjani amanabukeni lapho ingane inanekekelwa khona ngenkathi usemsebenzini (lokhu kungaba indawo la wena osebenzela khona, ikhaya lakho, ikhaya lombheki wakhe noma inkulisa)?**		
15.10	Is there electricity access where the child is cared for while you are working (this could be your own home, the carer's home or the crèche)? **Ingabe ukhona ugesi la ingane inakekelwa khona ngenathi usemsebenzini (lokhu kungaba indawo la wena osebenzela khona, ikhaya lakho, ikhaya lombheki wakhe noma inkulisa)?**	1 = Yes	0 = No
15.11	Is there access to a fridge where the child is cared for while you are working (this could be your own home, the carer's home or the crèche)? **Ingabe sikhona isiqandisi la umntwana anakekelwa khona ngenkathi usemsebenzini (lokhu kungaba indawo la wena osebenzela khona, ikhaya lakho, ikhaya lombheki wakhe noma inkulisa)?**	1 = Yes	0 = No
